# Hypertension Does Not Alter the Increase in Cardiac Baroreflex Sensitivity Caused by Moderate Cold Exposure

**DOI:** 10.3389/fphys.2016.00204

**Published:** 2016-06-02

**Authors:** Heidi E. Hintsala, Antti M. Kiviniemi, Mikko P. Tulppo, Heta Helakari, Hannu Rintamäki, Matti Mäntysaari, Karl-Heinz Herzig, Sirkka Keinänen-Kiukaanniemi, Jouni J. K. Jaakkola, Tiina M. Ikäheimo

**Affiliations:** ^1^Center for Environmental and Respiratory Health Research (CERH), University of OuluOulu, Finland; ^2^Medical Research Center Oulu (MRC Oulu), Oulu University Hospital and University of OuluOulu, Finland; ^3^Research Unit of Internal Medicine, University of OuluOulu, Finland; ^4^Research Unit of Medical Imaging, Physics and Technology, University of OuluOulu, Finland; ^5^Finnish Institute of Occupational HealthOulu, Finland; ^6^Research Unit of Biomedicine, University of OuluOulu, Finland; ^7^Biocenter of Oulu, University of OuluOulu, Finland; ^8^Department of Gastroenterology and Metabolism, Poznan University of Medical SciencesPoznan, Poland; ^9^Center for Life Course Health Research, University of OuluOulu, Finland; ^10^Unit of Primary Health Care, Oulu University HospitalOulu, Finland; ^11^Internal Medicine, Oulu University HospitalOulu, Finland

**Keywords:** hypertension, cold temperature, baroreflex, blood pressure, variability

## Abstract

Exposure to cold increases blood pressure and may contribute to higher wintertime cardiovascular morbidity and mortality in hypertensive people, but the mechanisms are not well-established. While hypertension does not alter responses of vagally-mediated heart rate variability to cold, it is not known how hypertension modifies baroreflex sensitivity (BRS) and blood pressure variability during cold exposure. Our study assessed this among untreated hypertensive men during short-term exposure comparable to habitual winter time circumstances in subarctic areas. We conducted a population-based recruitment of 24 untreated hypertensive and 17 men without hypertension (age 55–65 years) who underwent a whole-body cold exposure (−10°C, wind 3 m/s, winter clothes, 15 min, standing). Electrocardiogram and continuous blood pressure were measured to compute spectral powers of systolic blood pressure and heart rate variability at low (0.04–0.15 Hz) and high frequency (0.15–0.4 Hz) and spontaneous BRS at low frequency (LF). Comparable increases in BRS were detected in hypertensive men, from 2.6 (2.0, 4.2) to 3.8 (2.5, 5.1) ms/mmHg [median (interquartile range)], and in control group, from 4.3 (2.7, 5.0) to 4.4 (3.1, 7.1) ms/mmHg. Instead, larger increase (*p* < 0.05) in LF blood pressure variability was observed in control group; response as median (interquartile range): 8 (2, 14) mmHg^2^, compared with hypertensive group [0 (−13, 20) mmHg^2^]. Untreated hypertension does not disturb cardiovascular protective mechanisms during moderate cold exposure commonly occurring in everyday life. Blunted response of the estimate of peripheral sympathetic modulation may indicate higher tonic sympathetic activity and decreased sympathetic responsiveness to cold in hypertension.

## Introduction

It is well-recognized, that winter season and cold temperature increase morbidity and mortality, mainly related to cardiovascular and respiratory causes (The Eurowinter Group, 1997; Mercer, 2003; Mourtzoukou and Falagas, 2007; Bhaskaran et al., 2009). Exposure to cold temperature elevates sympathetic activity and blood pressure (BP), induces hemoconcentration, increases cholesterol, fibrinogen, and erythrocyte counts (Mercer, 2003; Cuspidi et al., 2012), which all are risk factors for cardiovascular events. Respiratory infections, such as common cold, pneumonia and influenza, occur with peak incidence during winter (Mourtzoukou and Falagas, 2007), which may also trigger cardiac events. All these factors contribute to the increased cold related cardiovascular morbidity, physiologic mechanisms of which remain to be fully established. It has been also recognized, that, cold season aggravates the symptoms and course of hypertension (Cuspidi et al., 2012; Ikäheimo et al., 2013). It is possible that impaired BP regulation, such as lower baroreflex sensitivity (BRS) (La Rovere et al., 2008; Kiviniemi et al., 2014b) and larger BP variability (BPV; Parati et al., 2013), together with higher BP could also contribute to the risk of cardiovascular events among hypertensive people during winter. However, the effects of cold weather on short-term BP regulation in hypertensive population are not known.

Cold exposure induces a sympathetic vasoconstriction (Kellogg, 2006) and therefore an immediate increase in arterial BP by 10–30 mmHg as an average or even by 60 mmHg in some individuals (Hintsala et al., 2014a). Facial cooling causes a simultaneous vagal activation reducing heart rate (HR) and increasing HR variability (HRV) (Hintsala et al., 2014b), which may serve as a protective cardiovascular response and limit BP elevation.

The baroreflex is the dominant short-term control mechanism for BP (La Rovere et al., 2008). It buffers changes in BP by altering the activation level of vagal cardio-inhibitory neurons and sympathetic neurons both to the heart and peripheral blood vessels. Decreased BRS is associated with cardiovascular diseases, such as hypertension, and poor prognosis (La Rovere et al., 2008; Kiviniemi et al., 2014b). Impaired BRS could disturb BP regulation during short-term cold exposure and therefore increase the cold-related risk of cardiovascular events among hypertensive people. Studies among healthy individuals have shown an increased BRS during the cold face test (Eckberg et al., 1984; Hilz et al., 1999; Stemper et al., 2002) as well as during cold exposure without cooling of the head (Yamazaki and Sone, 2000; Mourot et al., 2007). The detected higher BRS in cold probably originates from the central vagal activation (Eckberg et al., 1984; Yamazaki and Sone, 2000; Stemper et al., 2002). This response could be blunted in hypertension, in which sympathovagal balance is commonly altered (Parati and Esler, 2012).

Short- and long-term BPV increases proportionally with BP and is independently associated to cardiac and vascular damage and incidence of cardiac events (Parati et al., 2013). Low frequency (LF) BPV is coupled with efferent synchronous oscillations and is enhanced during sympathetic stimulation, and thus considered as a surrogate measure of vascular sympathetic activity (Julien, 2006). Short-term cold exposure induces sympathetic vasoconstriction, during which we could therefore expect increased BPV, but results from previous experimental studies are contradictory (Yamazaki and Sone, 2001; Stemper et al., 2002). Epidemiological studies, instead, have shown an inverse relation between 24 h BPV and environmental temperature (Winnicki et al., 1996; Jehn et al., 2002). To our knowledge, no previous experimental studies have assessed BPV in cold among hypertensive subjects.

The aim of the present comprehensive experimental study was to assess the effect of short-term cold exposure on spontaneous cardiac BRS and BPV among untreated hypertensive middle-aged men. Our hypotheses were that (1) cold exposure stimulates cardiac BRS and BPV and (2) this response is blunted in hypertension. For this purpose we performed a controlled experiment resembling habitual winter season exposure in the subarctic environment.

## Materials and methods

### Subjects

We conducted a population-based recruitment in 2011 which has been previously described in detail (Hintsala et al., 2014a). Briefly, a random sample of 1000 men aged 55–65 years and living in the city of Oulu (65°N, 25°E) were drawn from the Finnish Population Register. Half of them were reached and interviewed for eligibility and 204 performed home BP measurements for a week according to the recommendations of the European Society of Hypertension (Parati et al., 2008). Based on the home measurements subjects were classified as hypertensive (systolic BP ≥135 and/or the diastolic BP ≥85 mmHg) or men without hypertension, control group, (systolic/diastolic BP < 135/85 mmHg). A total of 89 men went through the exposure measurements in the climatic laboratory. The exclusion criteria were the presence of coronary heart disease or respiratory disease, use of antihypertensive drugs, an average home BP ≥175/105 mmHg, no home BP measurements, inadequate continuous BP data quality in the laboratory measurements (*n* = 42; automatic calibration >2 times/min, frequent arrhythmias, or noisy signal), and having a respiratory infection a week before exposure measurements. According to drop-out analysis, there were no differences between the subjects included to the final analyses and the original sample attending to the laboratory measurements. The final study group consisted of 24 hypertensive men and 17 men in control group (Table 1). The clinical characteristics did not differ among the groups, except three hypertensive subjects had type 2 diabetes and HR was higher and physical fitness lower in hypertensive compared to control group. The study was approved by the ethics committee of Northern Ostrobothnia Hospital District (EETTMK: 111/2010) and all participants of the study gave written informed consent. The study has been registered in the ClinicalTrials registry (www.clinicaltrials.gov, ID: NCT02007031).

**Table 1:** **Characteristics of the study group**.

**Variable**	**Hypertensive, *n* = 24**	**Controls, *n* = 17**	***P*-values**
Age, years	60 (59–61)	61 (59–62)	0.45
Height, cm	176 (174–179)	175 (172–178)	0.48
Weight, kg	86 (82–89)	80 (75–86)	0.09
BMI, kg/m^2^	27 (26–29)	26 (25–28)	0.12
BF, %	26 (23–28)	24 (21–26)	0.24
SBP, mmHg	142 (139–146)	119 (115–124)	−
DBP, mmHg	86 (83–89)	74 (71–77)	−
HR, bpm	69 (66–72)	64 (61–67)^*^	0.04
Estimated VO2max, ml/kg/min	35 (32–37)	38 (35–42)^*^	0.04
Diabetes mellitus, *n* (%)	3 (13)	0 (0)	−
Ever smoker, *n* (%)	10 (42)	4 (24)	0.32
Alcohol consumption ≥1 time/month, *n* (%)	18 (75)	14 (82)	0.71

*Continuous variables are presented as mean values and 95% confidence intervals, categorical variables as number of cases and percentages. BMI, body mass index; BF, body fat percentage; SBP, systolic blood pressure in home measurements; DBP, diastolic blood pressure in home measurements; HR, heart rate in home measurements; and estimated VO2max, indirectly estimated maximal oxygen uptake*.

* *p < 0.05 vs. hypertensive (group), assessed with independent t-tests and chi-square tests*.

### Experimental protocol

The experimental measurements were performed in autumn 2011 during office hours by trained professionals. The experiments began with a short introduction of the measurement protocol and visit to the climatic chamber. Height was measured. Body composition was assessed by bioelectrical impedance analysis (InBody 720; Biospace, Seoul, Korea) and physical fitness was estimated from resting HR and HRV (Crouter et al., 2004) (Polar S610; Polar, Kempele, Finland). The study subjects were equipped with skin temperature thermistors, electrocardiogram (ECG) electrodes, a cuff for measuring brachial BP, and equipment for continuous BP measurements, and dressed with three-layer winter clothing (ca. 2 clo during cold exposure, including hat and gloves, and 1.6 clo during baseline measurements (ISO 9920, 2007). The exposure protocol consisted of two consecutive 15 min phases during which the participants were standing with their arms supported at the level of the heart: baseline measurements in a climatic chamber (air temperature of 18°C, air velocity < 0.3 m/s, and relative air humidity of 30%) and cold exposure in an adjacent wind tunnel (−10°C, 3 m/s, and 50%, respectively). We applied standing posture, because the exposure was designed to simulate habitual winter time exposure. Also, non-invasive measurements of spontaneous BRS have better reproducibility (Herpin and Ragot, 1997) and may better represent the physiological function of baroreceptors (Taylor and Eckberg, 1996) in standing than supine position.

### Baroreflex sensitivity and blood pressure variability

Three-lead ECG measurements (Cardiolife Tec-7100; Nihon Kohden, Tokyo, Japan) were performed throughout the protocol. Continuous arterial BP was measured from finger with a non-invasive method applying the volume clamp methodology (Imholz et al., 1998) (Nexfin; BMEYE Medical systems, Amsterdam, the Netherlands). A height sensor was placed on the level of the heart. Physiocal-calibration function was applied to maintain the level of continuous BP. Brachial BP was measured by automatic oscillometric BP monitor (Schiller BP-200 Plus; Schiller, Baar, Switzerland) at 3-min intervals during baseline and cold. Respiration frequency was measured with a piezoelectric belt (PneumoTrace; ADInstruments, Sydney, Australia). After the baseline period continuous BP measurement was discontinued for 2 min to normalize peripheral circulation. Signal recordings were carried out with the Power Lab/8SP (ADInstruments, Sydney, Australia) with 1 kHz sampling frequency and operated with LabChart 7.3.2 (ADInstuments, Sydney, Australia). The continuous BP was recalibrated according to brachial BP.

Data analyses were performed with custom-made Matlab-based (MathWorks; Natick, MA, USA) software. Abnormally shaped and ectopic beats were replaced with local average. Physiocal-calibration sequence was replaced by linear interpolation (Kiviniemi et al., 2014a). Time series of RR-interval and beat-to-beat systolic BP were extracted from the continuous ECG and BP to describe variability of values. Total BPV was estimated from standard deviation of systolic BP from data without detrending. For the spectral analysis, very low frequencies (< 0.04 Hz) of the RR-interval and systolic BP recordings were detrended by Savitzky-Golay method. Spectral estimates for LF (0.04–0.15 Hz) and high frequency (HF) (0.15–0.4 Hz) HRV and BPV were computed for the last 5 min before (baseline) and during cold exposure by applying Fast Fourier Transform (Welch's method, length of sequence 128 with 50% overlapping). Respiratory frequencies were computed by relation of respiration cycles and time. BRS was estimated by frequency domain analysis of RR-interval and systolic BP spectral values for LF band using alpha (α) method, which estimates cardiac BRS as square root of the ratio of RR-interval and systolic BP. The alpha method is based on a high degree linear correlation of RR-interval and systolic BP at LF band, with an assumption this relation of RR and systolic BP is caused by baroreflex (Pagani et al., 1988). Therefore, if coherence between RR and systolic BP on LF band was found < 0.5 this data was excluded from further analyses. HF band was not analyzed, as it represents respiratory sinus arrhythmia, and does not allow to distinguish baroreflex control of BP from other central activity (Taylor and Eckberg, 1996).

### Skin temperatures and thermal sensations

Skin temperature was measured with thermistors (NTC DC95; Digi-Key, Thief River Falls, MN, USA) and recorded at 12 s intervals with an eight channel temperature data logger (SmartReader Plus; Acr Systems, Surrey, Canada). The thermistors were placed on the middle finger, shoulder blade, and cheek. Thermal perception for the face and whole body was assessed using subjective judgement scale (ISO 10551, 1995).

### Statistical methods

The characteristics of the study subjects were compared between the study groups and the statistical significance for the differences between the groups was assessed by independent *t*-test for continuous variables and chi-square test for categorical variables (hypertensive vs. control group and for drop out analyses the original study sample vs. the subjects included to the final analyses). Cardiovascular parameters with a non-Gaussian distribution were transformed into natural logarithm for parametric statistical tests. The differences in the means between baseline and cold, as well as study groups were compared by two-way repeated measures analysis of variance and contrast tests. Sensitivity analyses were conducted by repeating the analyses without diabetic subjects (*n* = 3). Due to lack of effect to the results these were ignored in further analyses. Spearman (skewed variables) or Pearson correlation analyses were applied to assess relation between the parameter with group^*^time interaction and other measured parameters. The results are presented as medians and interquartile range (Q1, Q3) for skewed variables and as means and their 95% confidence intervals for variables with Gaussian distribution. Statistical analyses were performed with IBM SPSS for Windows version 20 (IBM Corp. Released 2011, Armonk, NY, USA) and significance was set at *p* < 0.05.

## Results

### Thermoregulatory responses

The employed cold exposure involved a rapid (from 30 to 22°C in 2 min) and robust (from 30 to 15°C in 10 min) reduction in facial skin temperature (cheek). The winter clothing prevented superficial cooling of most areas of the body, as demonstrated by a modest change in shoulder blade and finger temperature (shoulder blade; last 5 min of baseline 34°C and cold 31°C, and finger; last 5 min of baseline 28°C and cold 24°C). Thermal perceptions (median) of the face and whole body were neutral in baseline and cold (face) or cool (whole body) during last 5 min of the exposure. All thermoregulatory responses were comparable between the test groups.

### Baroreflex sensitivity and respiration

There was no significant difference in BRS level between the study groups. Also, cold exposure increased BRS with comparable changes in both test groups (Table 2, Figure 1). HRV increased and HR decreased in cold without group differences in the level or response. Respiration frequency did not change in cold (0.31 ± 0.08 and 0.30 ± 0.10 Hz in hypertensive men, 0.26 ± 0.05 and 0.24 ± 0.05 Hz in the control group during the baseline and cold measurements, respectively, *p* = 0.2).

**Table 2:** **Baroreflex sensitivity and blood pressure variability before (baseline) and during cold exposure**.

	**Hypertensive (*n* = 24)**			**Controls (*n* = 17)**		
**Variable**	**Baseline(Median, Q1, Q3, or Mean, 95% CI)**	**Cold (Median, Q1, Q3, or Mean, 95% CI)**	**Difference (Median, Q1, Q3, or Mean, 95% CI)**	**Baseline (Median, Q1, Q3, or Mean, 95% CI)**	**Cold (Median, Q1, Q3, or Mean, 95% CI)**	**Difference (Median, Q1, Q3, or Mean, 95% CI)**
LF BRS, ms/mmHg	2.6 (2.0, 4.2)	3.8 (2.5, 5.1)^*^	0.7 (0.1, 2.2)	4.3 (2.7, 5.0)	4.4 (3.1, 7.1)^*^	0.2 (−0.9, 2.9)
SBP SD, mmHg	8.8 (8.3, 9.8)	9.1 (7.2, 11.3)	0.4 (−1.9, 2.1)	6.8 (5.8, 8.0)^**^	7.9 (7.1, 9.7)^*^^,^^**^	1.5 (−0.4, 2.1)^#^
LF BPV, mmHg^2^	27 (19, 39)	27 (19, 51)	0 (−13, 20)	13 (6, 18)^**^	20 (13, 27)^*^	8 (2, 14)^#^
HF BPV, mmHg^2^	7 (4, 9)	10 (5, 21)^*^	3 (−1, 9)	4 (2, 8)	8 (5, 9)^*^	4 (0, 6)
LF RR, ms^2^	220 (130, 470)	330 (170, 590)^*^	110 (50, 290)	120 (80, 370)	280 (180, 1070)^*^	140 (50, 470)
HF RR, ms^2^	30 (20, 70)	80 (40, 210)^*^	60 (20, 170)	30 (20, 60)	130 (70, 240)^*^	90 (30, 200)
LF/HF ratio	7.5 (3.0, 12.4)	4.6 (2.4, 5.9)^*^	−2.2 (−6.6, −0.4)	5.2 (2.3, 8.3)	2.9 (2.2, 4.4)^*^	−1.3 (−3.4, 0.3)
HR, bpm	83 (79–88)	77 (72–82)^*^	−6 (−8–−4)	77 (69–85)	72 (65–78)^*^	−5 (−9–−1)
SBP, mmHg	149 (143–154)	178 (170–185)^*^	29 (23–35)	129 (123–135)^**^	157 (149–165)^*^^,^^**^	28 (21–34)
DBP, mmHg	94 (90–97)	105 (100–110)^*^	11 (8–15)	83 (77–88)^**^	94 (88–99)^*^^,^^**^	11 (8–14)

*Values are group medians and interquartile ranges (Q1, Q3) for skewed spectral estimates and group means and 95% confidence intervals for other parameters. Values were computed over 5 min phases in the end of baseline and cold exposure. In addition, the difference between cold and baseline is presented with medians and Q1, Q3, or means and 95% CI. BRS, baroreflex sensitivity; SBP, systolic blood pressure; SD, standard deviation; BPV, (systolic) blood pressure variability; LF, low frequency; HF, high frequency; RR, RR interval; HR, heart rate; DBP, diastolic blood pressure*.

* *p < 0.05 vs. baseline (time)*,

** *p < 0.05 vs. hypertensive (group)*,

# *p < 0.05 vs. (cold-baseline) difference among hypertensive (interaction), assessed with Two-way repeated measures analysis of variance and contrast tests*.

**Figure 1:**
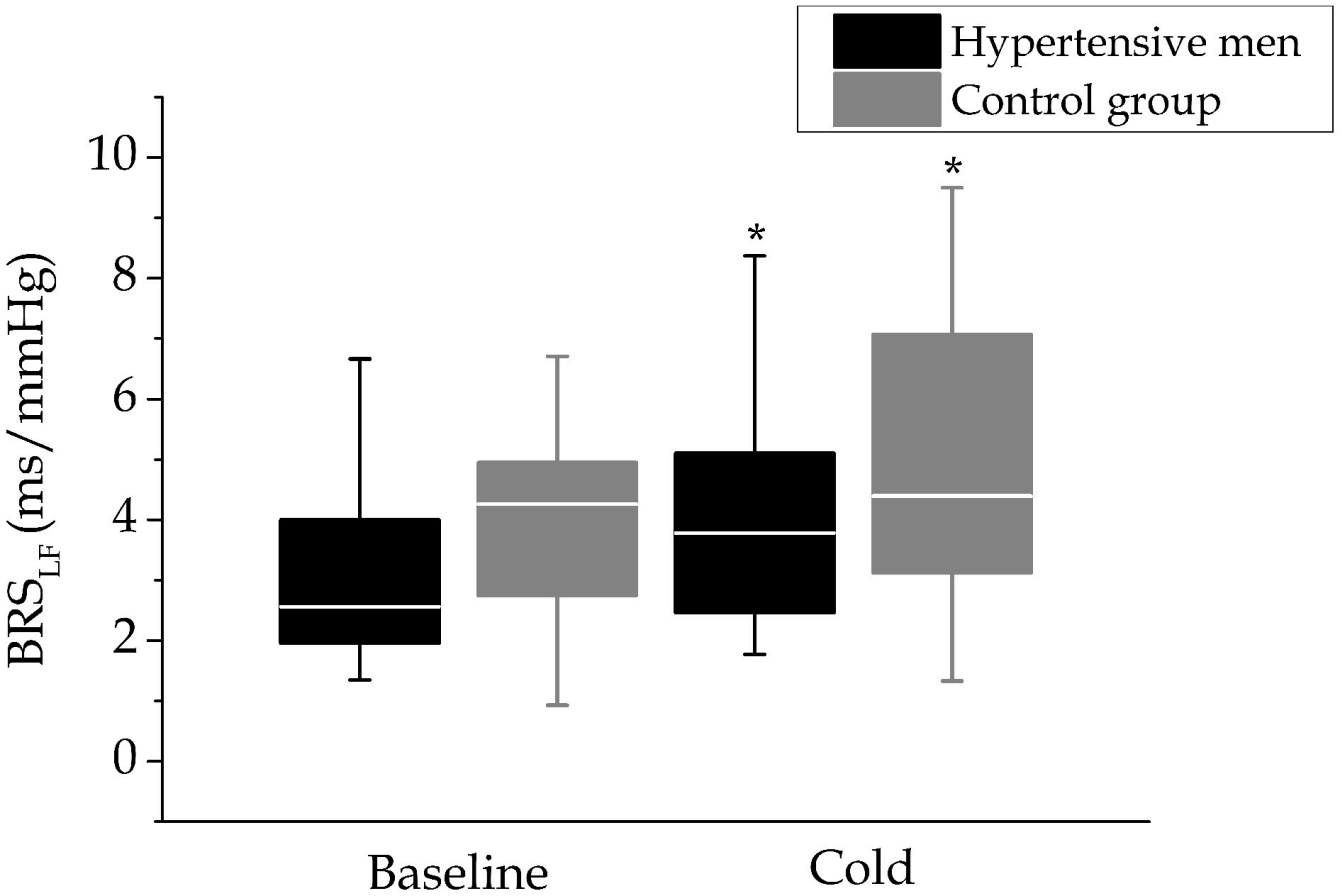
**Baroreflex sensitivity in cold**. Baroreflex sensitivity before (baseline) and at the end of the cold exposure in hypertensive men (*n* = 24) and control group (*n* = 17) depicted with boxplot figures. Baroreflex sensitivity level and changes in cold did not differ between the study groups. ^*^p < 0.05 vs. baseline (time).

### Blood pressure variability

**Figure 2:**
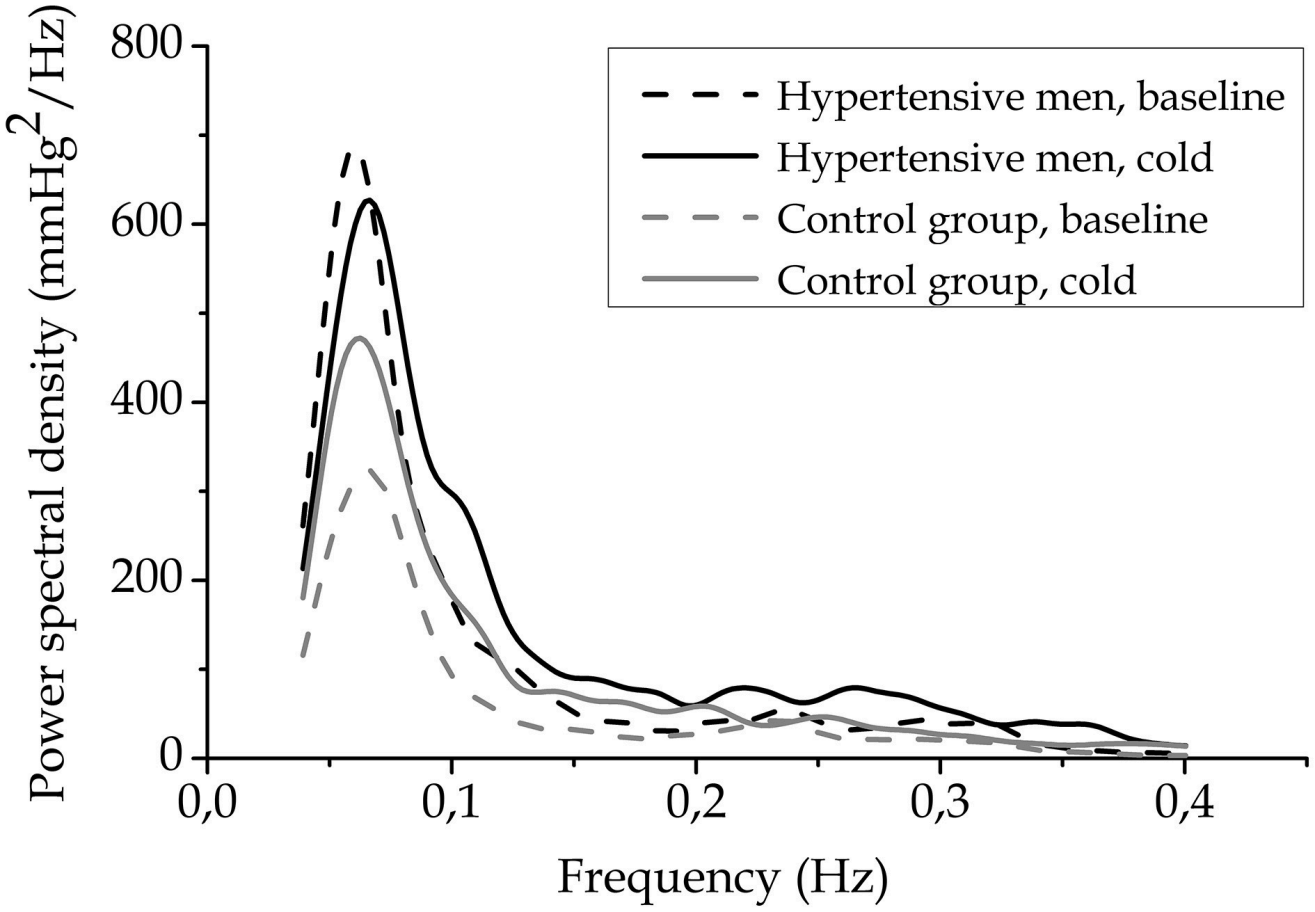
**Blood pressure variability spectra**. Blood pressure variability spectra for LF and HF bands before (baseline) and in the end of cold exposure as groups averages (*n* = 24, hypertensive men, and *n* = 17, control group).

LF, but not HF, BPV was significantly (*p* < 0.05) higher in hypertensive men than in the control group. HF BPV was increased during cold exposure in all, but LF BPV only in the control group (Table 2, Figure 2, interaction, *p* = 0.012). Results of total BPV were comparable to the ones detected on LF band. The interaction effect was not detected with cardiac (HRV) regulation. Higher cold induced response in LF BPV related to lower baseline LF BPV (Spearman correlation coefficient = −0.33, *p* = 0.04). LF BPV response did not correlate with baseline BRS or HRV.

## Discussion

Our novel findings provide evidence that cold exposure, resembling everyday winter circumstances in a subarctic climate, increases cardiac BRS, indicating increased vagal activity, similarly in hypertensive and control subjects. In addition, we showed for the first time that hypertension blunts the cold-induced increase in LF BPV, an index of vascular sympathetic modulation, observed in the control group.

The observed augmented cardiac BRS is consistent with previous studies among healthy subjects, which applied cold face test (Eckberg et al., 1984; Hilz et al., 1999; Stemper et al., 2002), cold water immersion (Mourot et al., 2007), or water-perfusion suit (Yamazaki and Sone, 2000, 2001) as forms of exposure. Estimation of BRS was performed either with neck suction (Eckberg et al., 1984), sequence method (Yamazaki and Sone, 2000; Mourot et al., 2007) or transfer function gain (Hilz et al., 1999; Stemper et al., 2002). In contrast to these studies, Cui et al. (2007), who assessed sympathetic vascular BRS through drug induced changes in diastolic BP and muscle sympathetic nerve activity, found an unaltered BRS slope during cooling with a water-perfusion suit. We observed that LF BPV increased in the cold among control subjects. In contrast to our findings, LF BPV did not change during a cold face test applying different breathing patterns (Stemper et al., 2002), but decreased during skin surface cooling without facial exposure (Yamazaki and Sone, 2001). In summary, the seemingly contradictory findings probably relate to the variation in the type of exposure (duration, intensity, exposed parts of the body), varying study settings (posture, breathing pattern), measurement (e.g., direct vs. indirect assessment) and computation methods (e.g., time or frequency domain), as well as individual characteristics (age). This highlights the importance of applying realistic cold exposure (standing or exercising, winter clothes, cold air exposure) with a population sample when the study is aimed to assess physiological links between habitual cold exposure and cardiovascular function.

In our study, cold exposure involving mainly facial cooling resulted in autonomic co-activation as judged by simultaneous rise of BP and HRV, and decrease in HR (Hintsala et al., 2014b). This has been previously shown with cold water immersion (Shattock and Tipton, 2012), whole-body cooling (Mäkinen et al., 2008) or cold face test (Tulppo et al., 2005). The observed increased BRS in cold has been suggested to result either from direct central vagal activation caused by cold exposure (Stemper et al., 2002) or as central interaction of signaling from baroreceptors and skin cold receptors (Eckberg et al., 1984; Yamazaki and Sone, 2000). A measure of peripheral sympathetic modulation, LF BPV, generally augments with sympathetic stimulus (Julien, 2006) and is inversely related with the level of BP (Mancia et al., 1983; Parati et al., 2013). The observed cold induced augmented LF BPV among control subjects is consistent with this theory. Data on cardiac pacing has shown that respiratory sinus arrhythmia generates respiratory-frequency (i.e., HF) fluctuation in arterial pressure (Taylor and Eckberg, 1996). This probably explains the observed augmentation of HF BPV simultaneously with a robust cold related increase in HF HRV, while respiration remained unaltered.

Hypertension is related to increased sympathetic and reduced vagal activity (Parati and Esler, 2012) and impaired BRS (La Rovere et al., 2008). Therefore, we hypothesized that vagal BRS responses to cold could also be blunted in hypertension. Contrary to our hypothesis, cold-related cardiac BRS and HRV responses were both comparable between hypertensive and control subjects. The lack of difference in BRS responses between the study groups in cold may be due to a short disease history among the untreated hypertensive subjects in our study. In fact, the observed higher HR among untreated hypertensive men resembles the hyperkinetic stage detected early, where BP is at a higher level due to increased cardiac output (Palatini and Julius, 2009). We also observed that hypertensive subjects did not show a cold-induced increase in LF BPV, in contrast with the controls. Comparable results were shown with total BPV. We speculate that the blunted response of BPV among hypertensive men in our study relates to chronically higher level of sympathetic activity among hypertensive subjects and indicates either saturation of sympathetic vascular oscillatory activity or downregulation of sympathetic responsiveness at central or vascular level. Previously, tonic sympathetic activity has been suggested to even abolish LF BPV among patients with advanced cardiovascular disease (van de Borne et al., 1997). Among hypertensive subjects, blunted LF BPV response has previously been detected during postural tilt (Aono et al., 1996). Charkoudian et al. (2006) have shown an inverse relation of vascular adrenergic responsiveness and tonic activity of sympathetic vasoconstrictor nerves in healthy humans. In our study, cold induced changes in BP were comparable between test groups (Hintsala et al., 2014a), which would support the possibility that blunted response is a sign of saturation of oscillatory vascular sympathetic activity.

The strength of our study is that all subjects were randomly drawn from the general population and thus the results reflect typical cardiovascular response to cold among untreated middle aged men with or without hypertension. Persons with untreated hypertension, or unaware of their condition, form a significant population: less than half of hypertensive persons take antihypertensive drugs (Wolf-Maier et al., 2003). Furthermore, we were able to produce a strictly controlled and equal cold exposure to all subjects. Hence, we consider that our results have public health implications due to the population-based sample and the utilized cold exposure which was similar to everyday winter circumstances in a cold climate. One methodological limitation of our study was the challenge to measure continuous BP from finger during cold exposure due to vasoconstriction. To overcome this, we applied automatic calibration of continuous BP signal throughout the experiments and adjusted the BP level according to brachial measurements. To avoid possible remaining bias, we excluded all data with inadequate quality according to pre-defined criteria. In addition, this limitation should not affect the group comparison, which is the main focus of our study. Also, vascular sympathetic activity was estimated indirectly from LF BPV. Direct measurement of muscle sympathetic nerve activity with microneurography would have provided more insight to the sympathetic vascular regulation. The results of our study are applicable to middle-aged men with normal BP or mild to moderate untreated hypertension. It must be noticed, that the results could significantly differ among older or younger subjects, women, those with more severe hypertension, or patients being treated with antihypertensive drugs, or having a cardiac disease, such as heart failure or coronary artery disease.

In conclusion, our results demonstrate that moderate short-term whole body cold exposure, a type of exposure that occurs during occupational and leisure time activity in subarctic countries (Mäkinen et al., 2006), temporarily increases cardiac BRS in middle-aged men. These changes are comparable between men with or without hypertension, which suggests that despite of the increased BP in cold, cardiovascular protective mechanisms are not disturbed in mild hypertension. However, LF BPV, an estimate of sympathetic vascular modulation, increased in control group but not hypertensive men who may experience a higher tonic sympathetic activity. Increased morbidity and mortality from cardiovascular causes in winter underlines the need for understanding the pathophysiological responses which are likely to contribute to these adverse health effects. Clinically, information related to cardiovascular regulation and responses in cold weather at the early stages of hypertension would be relevant for secondary prevention (e.g., advice on proper cold protection). Although, our results do not suggest that regulation of BP is impaired in cold weather among persons with mild hypertension, further research is needed among elderly people and patients with more advanced cardiovascular disease, such as more severe hypertension or cardiac disease.

## Author contributions

HH design of the study, acquisition, analysis, and interpretation of data, drafting, and final approval of the manuscript and its integrity. AK design of the study, acquisition, analysis, and interpretation of data, drafting, and final approval of the manuscript and its integrity. MT design of the study, acquisition, analysis, and interpretation of data, drafting, and final approval of the manuscript and its integrity. HH acquisition, analysis and interpretation of data, drafting, and final approval of the manuscript and its integrity. HR design of the study, acquisition and interpretation of data, drafting and final approval of the manuscript and its integrity. MM design of the study, interpretation of data, revision, and final approval of the manuscript and its integrity. KH design of the study, interpretation of data, revision, and final approval of the manuscript and its integrity. SK design of the study, interpretation of data, revision, and final approval of the manuscript and its integrity. JJ design of the study, interpretation of data, drafting and final approval of the manuscript and its integrity. TI design of the study, acquisition and interpretation of data, drafting and final approval of the manuscript and its integrity.

## Funding

This work was supported by the National Institute for Health and Welfare (Finland); Ida Montin Foundation (Finland); Juho Vainio Foundation (Finland); and Finnish Foundation for Cardiovascular Research (Finland).

### Conflict of interest statement

The authors declare that the research was conducted in the absence of any commercial or financial relationships that could be construed as a potential conflict of interest.
